# A Geographic Information Systems (GIS)-based analysis of modern South African rodent distributions, habitat use, and environmental tolerances

**DOI:** 10.1002/ece3.384

**Published:** 2012-10-16

**Authors:** Timothy L Campbell, Patrick J Lewis, Monte L Thies, Justin K Williams

**Affiliations:** 2 Department of Biological Sciences, Sam Houston State UniversityBox 2116, Huntsville, Texas, 77341

**Keywords:** Distribution maps, environmental tolerances, GIS, rodents, South Africa, vegetation

## Abstract

Goals of this study were to: (1) develop distributional maps of modern rodent genera throughout the countries of South Africa, Lesotho, and Swaziland by georeferencing museum specimens; (2) assess habitat preferences for genera by cross-referencing locality position with South African vegetation; and (3) identify mean annual precipitation and temperature range where the genera are located. Conterminous South Africa including the countries of Lesotho and Swaziland Digital databases of rodent museum specimens housed in the Ditsong National Museum of Natural History, South Africa (DM), and the Division of Mammals, National Museum of Natural History, Smithsonian Institution, United States (NMNH), were acquired and then sorted into a subset of specimens with associated coordinate data. The coordinate data were then used to develop distributional maps for the rodent genera present within the study area. Percent habitat occupation and descriptive statistics for six climatic variables were then determined for each genus by cross-referencing locality positions with vegetation and climatic maps. This report presents a series of maps illustrating the distribution of 35 rodent genera based on 19,471 geo-referenced specimens obtained from two major collections. Inferred habitat use by taxon is provided for both locality and specimen percent occurrence at three hierarchical habitat levels: biome, bioregion, and vegetation unit. Descriptive statistics for six climatic variables are also provided for each genus based on locality and specimen percent incidence. As rodent faunas are commonly used in paleoenvironmental reconstructions, an accurate assessment of rodent environmental tolerance ranges is necessary before confidence can be placed in an actualistic model. While the data presented here represent only a subset of the modern geographic distributions for many of the taxa examined, a wide range of environmental regimes are observed, suggesting that more research is necessary in order to accurately reconstruct an environmental signature when these taxa are found in the fossil record.

## Introduction

Rodent fossils are found in many Plio-Pleistocene fossil-bearing localities within southern Africa (Winkler et al. 2010), and are often used for reconstructing past environments (e.g., Avery 1984, 1987, 1992a,b, 1995, 2001; Cartmill 1967; De Graaff 1960; Matthews et al. 2005, 2009; Thackeray 1987; Thackeray and Avery 1990). Rodent fossils are considered particularly informative in paleoenvironmental reconstructions due to their specious and near ubiquitous nature, small home range sizes for most taxa, and because some taxa demonstrate ecological specificity that can provide detailed information on such factors as vegetation, substrate type, and climatic conditions within a localized area (De Graaff 1981; Kingdon 1997; Nowak 1991; Roberts 1951; Skinner and Chimimba 2005; Smithers 1971).

Paleoenvironmental reconstructions utilizing fossil faunas as proxies for past environmental conditions are based on the principle of actualism, which assumes that environmental tolerances of extant taxa are similar to the fossil taxa they morphologically resemble (Evans et al. 1981; Wesselman 1984, 1995; Patnaik 2003 Stoetzel et al. 2007, 2011; Wesselman et al. 2009). As such, to accurately reconstruct past environments and avoid distorted paleoenvironmental signatures, comprehensive neontological data must be collected in order to accurately ascertain a taxon's fundamental niche, defined as the set of all ecological factors forming an n-dimensional hypervolume in which a taxon is potentially able to exist indefinitely (Hutchinson 1957). However, it follows that over the course of a taxon's survivorship, conditions controlling a taxon's biogeographic distribution may change and current factors influencing modern distributions may not be analogous to those of the past (Van Couvering 1980; Wesselman 1984, 1995; Andrews 1990; Aguilar et al. 1999; Patnaik 2003; Wesselman et al. 2009). Although various biotic and abiotic factors serve to limit a taxon to a smaller realized niche (Hutchinson 1957; Lomolino et al. 2006), without a detailed understanding of a taxon's modern ecological tolerances, paleoenvironmental reconstructions using modern faunas as proxies must be viewed with caution.

**Figure 1:**
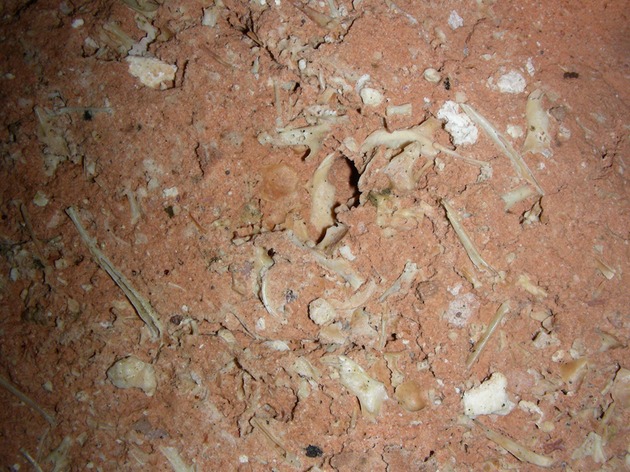
Microfaunal remains from the Koanaka Hills, Northwestern Ngamiland, Botswana.

This analysis attempts to improve our ability to reconstruct Plio-Pleistocene paleoenvironments in southern Africa by identifying habitat use and environmental tolerance ranges of extant rodents at the genus level, within the countries of South Africa, Lesotho, and Swaziland. In doing so, we utilize Geographic Information Systems (GIS) technologies and existing collections to quantify the number of individual specimens and unique localities within a hierarchical series of vegetation types. Museum specimens curated in the Ditsong National Museum of Natural History (DM; formerly the Transvaal Museum), South Africa, and the Smithsonian Institution's National Museum of Natural History (NMNH), Washington D.C. were used. Genus-level assessment was chosen for this analysis as this is generally the lowest common taxonomic level to which most micromammalian taxa can be unambiguously identified using skeletal remains (Fig. 1) (Reed 2007; Reed and Geraads 2012). Additionally, examinations of both modern and fossil specimens by several of the authors (TLC, PJL, MLT) from the Koanaka Hills in Botswana have suggested that without the utilization of molecular techniques, identification to the species level of many rodent taxa in the region should be avoided due to a lack of defined apomorphies (Lewis et al. 2011). By quantifying rodent genus-level distributions along with climate data and vegetation types for South Africa, Lesotho, and Swaziland, this study provides a conservative baseline from which actualistic models of past environments may be developed.

## Materials and Methods

Many of the methods used in this study to assess genus-level presence of various rodent taxa within vegetation units were previously outlined in a study of habitat use and environmental tolerances for southern African gerbils in the genus *Gerbilliscus* by Campbell et al. (2011). Although largely similar, key differences between this and the previous study include the hierarchical level at which vegetation data were assessed and the application of Google Earth™ for data validity assessment. Here, we review these methods and provide further details on these differences within their corresponding sections. Additionally, the effects of the differences between Campbell et al. (2011) and this present study are considered.

### Rodent distributional data

Rodent distributional data were obtained from electronic copies of the databases from the DM and the NMNH. Specimen designations were first screened and corrected to reflect current taxonomy following Wilson and Reeder (2005). The initial dataset for the study area obtained from the DM consisted of 17,815 museum records. Records that lacked latitude/longitude data, identical duplicate records, and records in which more than one taxon was assigned the same museum accession number were removed as it was not possible to identify the source of these discrepancies from the digital databases. This last step resulted in slightly different counts obtained for *Gerbilliscus* spp. than those reported by Campbell et al. (2011) as several specimens from different genera were found to have the same museum accession number as several specimens of *Gerbilliscus*. Records with latitudinal and longitudinal data reported in a quarter degree grid system were considered too coarse for this analysis and were also excluded, along with records for commensal, or introduced species. Records of genera found to be lacking a species designation with both an introduced and natural species (e.g., *Mus* spp.) were also removed, which resulted in a total of 12,383 DM records.

Latitudinal and longitudinal data were standardized into decimal degree (DD) format and projected in the Hartebeesthoek94 coordinate system as point data using ArcMap 10.0 (ESRI, Redlands, CA, 2010)**.** The decision to standardize point data using the Hartebeesthoek94 coordinate system was made with the understanding that some of the specimens' latitudinal and longitudinal data may have been recorded using alternate coordinate systems, including the Cape Datum system and the WGS84 reference system. Although a comprehensive vetting of the field notes associated with the collections may have resolved some of these issues, this was beyond the scope of this study. As the WGS84 coordinate was used in the calculations of the Hartebeesthoek94, these two systems are essentially compatible (Wonnacott 1999). For those specimens in which the original latitudinal and longitudinal data were recorded in the Cape Datum system, projection into the Hartebeesthoek94 coordinate system results in errors ranging between 20 and 90 m for longitude and 292 and 300 m for latitude (Wonnacott 1999), and were deemed acceptable for this study.

Museum data obtained from the NMNH consisted of 7982 records, all possessing latitudinal and longitudinal data. After removing commensal species and duplicate records as outlined above, a total of 7905 records were standardized into DD format and projected in the Hartebeesthoek94 coordinate system.

Once the rodent distributional data were projected, data validity was assessed by cross-referencing museum record provenance with that found on the base maps obtained from shapefiles provided in the latest treatment on the vegetation of South Africa, Lesotho, and Swaziland (Mucina and Rutherford 2006). Latitudinal and longitudinal data for points found outside geographic and geopolitical boundaries of the study area were first checked for data entry and conversion errors. Points still found outside the study area after corrections had been made were then examined individually. Here, several points were found to be either on or slightly over boundary lines (usually < 200 m). In these cases, straight-line distances were calculated from the projected points to the closest points within the study area and corresponding corrections were made to the latitudinal and longitudinal records. All points found to be outside the study area for which the provenance could not be reconciled were removed.

For the second round of data validity assessment, points were initially checked against aquatic features found within the study area including major rivers, lakes, and dams. Utilizing the methods outlined above, latitudinal and longitudinal data were first corrected for any entry errors and then straight line distances were calculated to remove the point from the aquatic feature. Following this, points containing state or provincial placement data were checked against geopolitical boundaries within the study area consisting of South African provincial borders and the state borders of Lesotho and Swaziland. Points found on or over state borders were checked and corrected using the methods outlined above. Assessment of data validity for points found on or within incorrect provincial borders proved slightly more difficult as numerous specimens were collected prior to the redesignation of South African internal geopolitical boundaries (Griggs 1995). Consequently, data accuracy was assessed for these points by comparing specific locality information provided in the museum records against infrastructural maps found in Mucina and Rutherford (2006) and by checking point provenance data against maps found on Google Earth™. This last step, the use of Google Earth™ for provenance assessment, departs from the methods used by Campbell et al. (2011). After removing all records with irreconcilable locality-specific provenances, a total of 19,471 rodent records were georeferenced as 1527 unique localities with 11,785 and 7686 of the records coming from the DM and NMNH, respectively. All subsequent analyses were run using both individual rodent records and individual localities in order to compare the values obtained. As the specimens used here were collected by numerous researchers at different times, different sampling strategies and research goals may have resulted in preferential collection of specific taxa at various localities, termed a collectors bias (Campbell et al. 2011). If such a collector bias has occurred, an analysis of the number of specimens within a vegetation unit would return inflated values not proportionate to the actual density of specimens in that habitat. Alternately, if a complete collection strategy was utilized, a greater number of specimens at a locality may indicate a greater abundance and thus a habitat preference. As it was not possible to evaluate the collection methodologies utilized in the acquisition of the specimens, and it remains unclear as to which digital sampling strategy is to be preferred, we provide both sets of data. When the values obtained using these different sampling strategies are largely congruent, greater confidence is gained in the calculated habitat signal for each taxon.

### Climate data

Climate data used in this analysis were obtained from the WORLDCLIM v. 1.4 database (http://www.worldclim.org) (Hijmans et al. 2005). WORLDCLIM is a set of global climate layers (grid data in raster format) available in spatial grids ranging in resolution from 30 arcsec to 10 arcmin at the equator. These spatial resolutions are approximately 0.86 km^2^ and 344 km^2^ at the equator, respectively. Long-term monthly precipitation and temperature averages are available from this database along with 19 bioclimatic variables derived from averaged monthly temperature and rainfall values. Of these, six were selected at the highest resolution level available for inclusion in this analysis: mean annual temperature (MAT), maximum temperature of warmest month (MxTWM), minimum temperature of coldest month (MnTCM), mean annual precipitation (MAP), precipitation of the wettest month (PWM), and precipitation of driest month (PDM). These six climatic variables were selected as they define not only the gross means but also the extreme means of the areas inhabited by the modern rodent taxa in the study area.

Once downloaded, selected climatic variables were projected using the Hartebeesthoek94 datum format. In order to efficiently combine the climatic raster data with the rodent point data, climatic rasters were converted to searchable polygons. Once converted using the latitudinal and longitudinal limits provided by the base maps, each of the climatic variables produced a unique number of searchable polygons with discreet climatic values. Localities containing rodent data were then referenced along with each climatic variable and all values were recorded.

### Vegetation data

Vegetation and geographic data used in this analysis were obtained, along with geopolitical and infrastructural data, from shapefiles provided in the latest treatment on the vegetation of South Africa, Lesotho, and Swaziland (Mucina and Rutherford 2006). This vegetation model is organized in a three-level nested hierarchy ranging from landscape scale vegetation units, to bioregions, and finally biomes. As large areas of land have been altered through anthropogenic influences such as farming and urban development, a mapping theme was largely adopted in which the vegetation model reflects the potential natural vegetation of the area mapped (Mucina and Rutherford 2006:15). Shapefiles of this vegetation model were projected in the Hartebeesthoek94 coordinate system, which was developed to refine older South African coordinate systems (Wonnacott 1999). Vegetation shapefiles covering an area of about 1.27 million km^2^, from around 22.13 to 34.83 decimal degrees (DD) South and 16.46 to 32.89 DD East were projected in ArcMap© v. 10.0 (ESRI, Redlands, CA, 2010).

To quantify rodent genus presence by vegetation type, a query was run for all localities for each vegetation unit. Percent occurrence by both number of specimens and number of localities containing each specific genus within each vegetation unit were then recorded, along with the corresponding biome and bioregion. Depending on the detail and quality of the data used in the construction of the vegetation shapefiles, precision down to 100 m and lower was possible in some areas (Mucina et al. 2006). This process differed from the study conducted by Campbell et al. (2011:49) in that the focal unit queried herein consisted of the individual vegetation units as opposed to individual biomes and bioregions. The rational for this difference was twofold. First, while remote sensing analyses at macrohabitat level resolutions may be unable to provide detailed information on rodent microhabitat use, it may be possible to gain an understanding of some particular ecological requirements for each taxon when all landscape scale vegetation unit descriptions are vetted. Second, during the construction of the biome and bioregion maps used here, biome polygons under 2000 hectares and bioregion polygons less than 600 hectares were excluded from the smaller scale maps and dissolved into adjacent, or surrounding units of the same level in order to avoid creating “salt and pepper patterns” when displaying a larger surface area (Mucina et al. 2006). As such, any specimen found within a lower level vegetation unit and queried at the higher biome level would return a habitat signature different from that of the corresponding vegetation unit in which it is located if this unit was dissolved based on polygon size. These limitations due to mapping scale, however, are eliminated by directly searching at the vegetation unit level and recording the corresponding biomes and bioregions in which that vegetation unit falls.

Finally, in this analysis, we follow Campbell et al. (2011) in considering vegetation units containing unique hydrogeological and pedological conditions influencing the local floristic composition as a separate higher order vegetation type from the biome in which they are embedded. As such, along with the nine biomes defined by Mucina and Rutherford (2006), we also include an Azonal biome in order to possibly identify additional factors associated with the various rodent genera distributions examined herein.

## Results

### Rodent distributions maps

**Figure 2:**
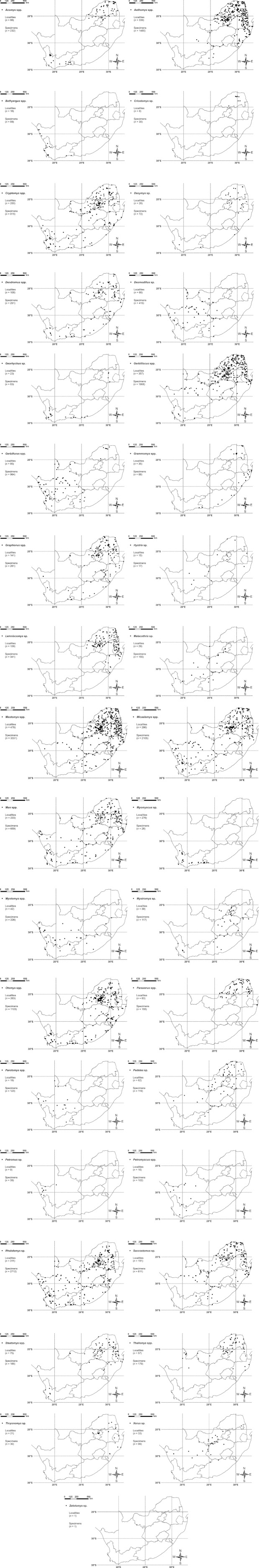
Distribution maps for 35 southern African rodent genera found within the countries of South Africa, Lesotho, and Swaziland as determined from museum specimen records.

Individual distribution maps for each of the 35 rodent genera are provided in alphabetic order by taxon in Fig. 2. The numbers of specimens and localities georeferenced for each taxon were found to be highly variable, ranging from 478 localities and 3331 specimens for *Mastomys* to 1 specimen and locality for *Zelotomys*. In total, the median number of localities for all taxa was 62 while the median number of specimens was 185.

### Rodent climate tolerances

Average values for the six climatic variables used here as determined for each taxon by number of specimens and number of localities are provided in Table 1. Although the average MAT occupied varied depending on method of calculation, the median temperature for both number of specimens and numbers of localities was 18.0°C. The range for average MAT occupied was found to vary the greatest when calculated by number of specimens, with the lowest values of 15.6°C obtained for *Myomyscus* and the highest values of 21.2°C obtained for *Paraxerus*. This trend of greater range in values obtained when calculated by number of specimens is also observed in the other five bioclimatic variables used here. In terms of MAP, median values of approximately 616 and 581 mm were obtained when calculated by number of localities and specimens, respectively. The lowest average MAP occupied was found to be 120 mm calculated for *Petromyscus* and the highest values calculated were 961 mm for *Grammomys*. Although averages are provided here for each taxon, it is felt that in order to better understand the environmental conditions possibly influencing a taxon's biogeography, it is better to consider the range on climatic values inhabited. As such, complete descriptive statistics for each variable can be found in the supporting online material (Appendix S1).

**Table 1:** Number of specimens (S) and number of localities (L) for 35 southern African rodent genera used to calculate average values for six climatic variables. Climate variables are as follows: mean annual temperature (MAT), maximum temperature of warmest month (MxTWM), minimum temperature of coldest month (MnTCM), mean annual precipitation (MAP), precipitation of the wettest month (PWM), and precipitation of driest month (PDM)

	Number of Specimens		MAT (°C)		MxTWM (°C)		MnTCM (°C)		MAP (mm)		PWM (mm)		PDM (mm)	
	
Taxon	Localities (L)	Specimens (S)	L	S	L	S	L	S	L	S	L	S	L	S
*Acomys*	66	232	19.0	18.3	29.8	29.3	5.2	4.9	559	556	101	95	9	12
*Aethomys*	339	1485	19.5	19.0	29.6	29.7	5.5	4.8	687	657	124	119	9	9
*Bathyergus*	18	89	17.0	17.3	27.5	27.4	7.1	7.4	383	355	61	53	12	14
*Cricetomys*	8	30	18.9	18.1	28.0	27.0	5.8	5.3	879	945	178	194	10	11
*Cryptomys*	200	915	17.6	17.7	28.6	28.3	3.5	3.3	666	710	116	126	10	10
*Dasymys*	26	72	18.7	18.7	28.3	28.3	5.6	5.5	802	801	137	148	13	12
*Dendromus*	108	251	17.6	17.4	27.8	27.1	4.5	5.2	749	825	125	132	14	17
*Desmodillus*	66	415	18.0	17.6	32.5	32.5	2.3	1.3	278	325	49	57	6	7
*Georhychus*	23	83	15.7	16.6	26.6	28.3	4.4	5.0	717	600	108	89	24	19
*Gerbilliscus*	357	1908	19.2	19.5	30.2	30.8	4.4	4.6	594	562	109	103	6	7
*Gerbillurus*	85	964	18.0	18.3	31.5	32.5	3.7	3.2	260	230	47	42	5	5
*Grammomys*	35	88	18.8	18.5	27.4	26.6	7.6	7.8	885	961	142	156	20	20
*Graphiurus*	141	261	17.9	17.5	28.1	27.8	4.0	3.9	715	724	126	125	10	11
*Hystrix*	15	17	18.1	18.1	30.5	30.8	3.0	2.8	491	478	87	85	8	8
*Lemniscomys*	139	341	19.8	19.9	29.9	30.0	5.7	5.7	682	676	123	123	8	8
*Malacothrix*	29	155	16.6	16.4	30.7	30.5	0.6	0.7	445	438	77	74	8	9
*Mastomys*	478	3331	18.5	18.1	29.0	28.8	4.3	3.9	695	708	123	123	9	10
*Micaelamys*	286	2105	18.2	18.1	29.7	30.8	3.9	3.3	565	441	102	79	7	7
*Mus*	233	659	18.0	17.7	28.7	28.4	4.3	4.6	665	692	112	110	12	15
*Myomyscus*	28	276	16.0	15.6	27.1	26.8	5.3	4.9	647	646	82	78	33	35
*Myotomys*	42	226	16.3	16.0	29.1	28.1	3.4	3.7	273	248	45	42	7	6
*Mystromys*	39	117	15.9	15.9	28.0	28.4	0.9	0.9	649	646	110	107	10	11
*Otomys*	263	1103	17.0	16.5	27.4	26.9	3.5	3.2	740	747	126	127	12	14
*Paraxerus*	63	155	20.6	21.2	30.9	31.5	6.3	7.0	577	549	111	106	5	5
*Parotomys*	19	123	17.6	17.6	31.9	30.8	2.7	4.3	198	184	37	33	5	4
*Pedetes*	62	119	18.1	17.9	30.1	30.0	2.4	1.9	528	536	97	98	5	5
*Petromus*	9	39	18.2	17.7	31.6	30.8	5.4	5.2	149	156	25	25	4	4
*Petromyscus*	16	102	17.8	20.6	32.2	36.0	3.5	4.9	207	120	34	22	7	4
*Rhabdomys*	311	2712	16.7	16.3	28.2	27.7	2.6	2.8	624	604	107	102	10	11
*Saccostomus*	191	611	19.8	19.6	31.0	31.2	5.3	5.2	580	552	103	97	9	10
*Steatomys*	75	185	19.1	19.1	29.5	29.5	5.1	5.1	682	704	123	130	8	9
*Thallomys*	57	179	19.6	19.5	30.5	31.3	4.5	3.4	616	527	115	97	7	5
*Thryonomys*	21	30	19.0	19.4	28.9	29.3	5.3	5.8	753	762	133	135	11	12
*Xerus*	33	99	17.4	17.6	31.1	31.4	0.8	0.4	451	463	79	80	6	6
*Zelotomys*	1	1	17.6	17.6	31.9	31.9	1	1	473	473	85	85	3	3

### Rodent vegetation occupation

Percent biome occupation for each taxon calculated by both total number of specimens and total number of localities is provided in Table 2. In general, differences between values calculated for biome percent occupation by number of specimens and number of localities averaged approximately 3.8% with a median value of 2%. Six taxa, however, were found to have differences in percent biome occupation in excess of 10% across a variety of biomes. These taxa include *Dendromus*, *Georychus*, *Micaelamys*, *Myomyscus*, *Parotomys*, and *Petromyscus*. With the exception of *Micaelamys*, all of these taxa had fewer than 110 localities and 280 specimens associated with them (Table 1 and 2). These discrepancies may result from either a few georeferenced localities containing many specimens or many individual specimens being georeferenced as unique localities. The correspondence between taxon percent occupations calculated by both methods further breaks down at the lower bioregion and vegetation unit levels (Appendix S2).

**Table 2:** Percent occurrence of 35 southern African rodent genera within 10 biome level units as calculated by number of specimens (S) and number of localities (L). Number of specimens and localities for each taxon can be found in Table 1. Abbreviated biomes are as follows: Albany Thicket (A-Thicket), Indian Ocean Costal Belt (IOCB), Nama-Karoo (N-Karoo), and Succulent Karoo (S-Karoo)

	A-Thicket		Azonal		Desert		Forests		Fynbos		Grassland		IOCB		N-Karoo		Savanna		S-Karoo	
										
Taxon	L	S	L	S	L	S	L	S	L	S	L	S	L	S	L	S	L	S	L	S
*Acomys*	1.5	1.3	4.5	2.2	–	–	3.0	3.0	21.2	29.7	1.5	0.4	–	–	–	–	68.2	63.4	–	–
*Aethomys*	0.3	1.2	1.8	1.9	–	–	1.0	3.5	–	–	23.1	15.9	2.9	1.5	0.9	5.3	74.6	66.0	–	–
*Bathyergus*	–	–	11.1	10.1	–	–	–	–	72.2	66.3	–	–	–	–	–	–	–	–	16.7	23.6
*Cricetomys*	–	–	–	–	–	–	–	–	–	–	–	–	–	–	–	–	100	100	–	–
*Cryptomys*	1.5	1.1	2.5	2.0	–	–	2.0	0.4	10.0	8.5	31.0	29.5	5.0	2.2	3.0	0.8	42.0	53.7	3.0	1.9
*Dasymys*	–	–	–	–	–	–	7.7	5.6	3.8	2.8	11.5	8.3	7.7	5.6	–	–	69.2	77.8	–	–
*Dendromus*	1.9	1.2	3.7	4.0	–	–	4.6	4.8	10.2	10.8	29.6	27.1	5.6	15.9	0.9	0.4	43.5	35.9	–	–
*Desmodillus*	4.5	3.1	15.2	16.4	1.5	0.2	–	–	1.5	0.7	9.1	12.5	–	–	31.8	41.9	22.7	18.6	13.6	6.5
*Georhychus*	8.7	3.6	8.7	16.9	–	–	4.3	1.2	56.5	69.9	21.7	8.4	–	–	–	–	–	–	–	–
*Gerbilliscus*	–	–	3.1	4.8	0.3	0.1	0.8	0.6	2.8	3.5	20.4	20.6	1.7	1.3	2.8	4.1	67.5	64.5	0.6	0.6
*Gerbillurus*	2.4	5.6	10.6	20.9	2.4	0.5	–	–	8.2	1.6	3.5	1.8	–	–	28.2	29.9	22.4	14.9	22.4	24.9
*Grammomys*	–	–	2.9	1.1	–	–	25.7	35.2	–	–	20.0	13.6	11.4	17.0	40.0	33.0	–	–	–	–
*Graphiurus*	2.1	4.6	1.4	3.8	–	–	6.4	8.4	2.8	1.5	27.0	27.2	1.4	2.3	1.4	1.9	55.3	47.1	2.1	3.1
*Hystrix*	–	–	–	–	–	–	–	–	6.7	5.9	20.0	17.6	–	–	20.0	17.6	53.3	58.8	–	–
*Lemniscomys*	–	–	1.4	1.2	–	–	1.4	1.5	–	–	10.1	10.6	0.7	1.2	–	–	86.3	85.6	–	–
*Malacothrix*	3.4	3.9	13.8	7.7	–	–	–	–	–	–	37.9	36.1	–	–	27.6	41.9	10.3	5.8	6.9	4.5
*Mastomys*	1.5	1.4	3.8	4.7	–	–	2.5	1.3	–	–	29.1	28.4	2.7	3.1	2.5	4.8	57.7	56.2	0.2	0.0
*Micaelamys*	1.0	0.6	2.1	2.9	1.4	0.7	0.7	0.1	7.3	9.1	22.7	16.0	–	–	7.7	28.5	51.0	31.7	5.9	10.3
*Mus*	1.7	1.2	2.6	3.8	1.3	0.5	3.9	6.5	7.3	7.6	25.8	22.9	3.0	7.0	4.3	6.7	46.4	38.7	3.9	5.2
*Myomyscus*	–	–	7.1	2.9	–	–	10.7	31.5	78.6	64.5	–	–	–	–	–	–	–	–	3.6	1.1
*Myotomys*	2.4	0.4	7.1	8.4	–	–	–	–	11.9	6.2	11.9	9.3	–	–	23.8	29.2	–	–	42.9	46.5
*Mystromys*	2.6	0.9	10.3	4.3	–	–	–	–	7.7	8.5	64.1	73.5	–	–	5.1	3.4	7.7	7.7	2.6	1.7
*Otomys*	1.5	0.9	2.7	1.8	–	–	3.4	4.8	11.4	13.6	38.0	47.0	4.2	3.2	1.1	0.5	36.9	27.3	0.8	0.9
*Paraxerus*	–	–	3.2	7.7	–	–	1.6	0.6	–	–	–	–	3.2	1.3	–	–	92.1	90.3	–	–
*Parotomys*	–	–	21.1	9.8	–	–	–	–	–	–	–	–	–	–	42.1	22.0	10.5	8.9	26.3	59.3
*Pedetes*	–	–	8.1	5.9	–	–	–	–	–	–	27.4	32.8	–	–	11.3	8.4	53.2	52.9	–	–
*Petromus*	–	–	–	–	11.1	7.7	–	–	–	–	–	–	–	–	22.2	23.1	–	–	66.7	69.2
*Petromyscus*	–	–	–	–	18.8	55.9	–	–	12.5	2.9	–	–	–	–	31.3	18.6	6.3	2.9	31.3	19.6
*Rhabdomys*	1.9	1.0	4.8	5.1	0.6	0.1	3.2	2.7	9.0	10.5	38.6	39.7	1.0	1.9	8.7	10.4	25.4	15.6	6.8	12.9
*Saccostomus*	1.6	1.3	9.4	14.7	–	–	2.6	1.1	0.5	0.3	5.2	5.6	4.2	1.8	5.2	11.6	70.7	63.3	0.5	0.2
*Steatomys*	–	–	2.7	1.1	–	–	–	–	5.3	7.6	21.3	18.4	1.3	0.5	–	–	68.0	70.3	1.3	2.2
*Thallomys*	–	–	7.0	2.8	–	–	1.8	0.6	–	–	7.0	4.5	1.8	2.2	1.8	0.6	80.7	89.4	–	–
*Thryonomys*	–	–	–	–	–	–	–	–	–	–	14.3	10.0	4.8	6.7	–	–	81.0	83.3	–	–
*Xerus*	–	–	3.0	1.0	–	–	–	–	–	–	24.2	21.2	–	–	21.2	16.2	45.5	57.6	6.1	4.0
*Zelotomys*	–	–	–	–	–	–	–	–	–	–	–	–	–	–	–	–	100	100	–	–

## Discussion and Conclusions

### Differences in results

To summarize the two major differences that exist between this study and that of Campbell et al. (2011): (1) habitat use for each genus was assessed at a lower level vegetation class, that of the vegetation unit; and (2) Google Earth™ was used in conjunction with the base map data for specimen and locality provenance assessment. The combined effects of these two factors resulted in a slight increase in the number of specimens due to a few localities with many specimens being included within this analysis. Alternately, when specimens with the same museum accession number across genera were removed, the result was a slight decrease in the number of localities due to several containing singular specimens. Finally, when individual vegetation units were queried, several specimens and localities not identified at a gross level resolution were found. Although the values for both number of specimens and number of localities are slightly different, no value was found to differ by more than 2.2% at the biome level (Table 2 here; Table 1 Campbell et al. 2011).

### Methodological issues and future use

Although the issue of different percent occupation values calculated by both total number of specimens and localities at the biome level is problematic for some rodent taxa, the high degree of correspondence of most taxa suggests that this method can be used with some confidence when large sample sizes are compiled. As this analysis only used specimen data from two repositories, only one of which is located within the study area (DM), the inclusion of additional specimen distributional data from other regional repositories may potentially improve the correspondence between these two values at the various hierarchical vegetation levels. This, however, will only become practical when additional repositories collection records are digitized and made available. Finally, future studies should also consider the effects of both collection and collector biases in their analyses and report both locality and specimen data (Campbell et al. 2011).

Although the use of GIS-based technologies represents an important new method for improving our understanding of the habitat use and climatic tolerances of various taxa, studies can only be as accurate and precise as the data available. In terms of the study presented here, it is possible that the long-term climate averages utilized may mask short-term climatic shifts that could have had an impact on habitats, and therefore rodent distributions during the time specimens were collected. These issues could be resolved through the development of similar climatic data at either annual or decadal scales. Similarly, the vegetation data available and used here were limited to a small subset of the entire range of many of the taxa examined. In order for a complete understanding of the habitat tolerances of these taxa to be obtained, vegetation data of comparable quality needs to be generated for other areas and disseminated in a similar format. Additionally, it is important to keep in mind that these data, while of high quality, may not accurately reflect the “real” condition on the ground at any one time due to anthropogenic influences, such as farming and urban development (Mucina et al. 2006:15).

Although the data presented here (quantified values for percent habitat occupation and associated climatic variable statistics) may be used in a variety of ways, the primary purpose of this study is to improve the use of rodent proxy data in paleoenvironmental reconstructions. In particular, the application of taxon percent habitat occupation data is thought to be potentially useful as a more objective method for distributing niche model values for use in generating cumulative taxonomic habitat indices (THI) (Campbell et al. 2011). While work is currently ongoing to improve the application of these data to the THI method, preliminary results have suggested that this approach may help improve our ability to accurately reconstruct past environments and avoid spurious paleoenvironmental signatures based on a lack of understanding of the modern biota.

## Biosketch

**Timothy L. Campbell** is a PhD student in the Department of Anthropology at Texas A&M University. His research interests include the assessment of the utility of rodent faunas as proxies for past environmental change and Plio-Pleistocene paleoenvironmental reconstruction of hominin-bearing deposits.

The focus of the research team includes improving reconstructions of past environments using faunal proxies and the modeling of modern faunal and floral distributions using GIS technologies.

Author contributions: T.L.C., P.J.L., M.L.T., and J.K.W. conceived the overall idea for the project with T.L.C., P.J.L., and M.L.T. focusing on the faunal and paleontological themes, and T.L.C. and J.K.W. focusing on the GIS component. Data were collected and analyzed by T.L.C. The writing was led by T.L.C. with all authors contributing to the final product.

## Supporting Information

**Appendix S1.** Descriptive statistics calculated for each taxon by number of localities and specimens for the six bioclimatic variables selected from Hijmans et al. (2005).

**Appendix S2.** Taxon percent occurrence calculated by number of localities and specimens within the various biomes, bioregions, and vegetation units proposed by Mucina and Rutherford (2006).
